# A systematic survey on the application of federated learning in mental state detection and human activity recognition

**DOI:** 10.3389/fdgth.2024.1495999

**Published:** 2024-11-27

**Authors:** Albin Grataloup, Mascha Kurpicz-Briki

**Affiliations:** Bern University of Applied Sciences, Technik und Informatik, Biel, Switzerland

**Keywords:** mental health, well-being, human activity detection, federated learning, data heterogeneity, personalization, distributed, privacy-preserving

## Abstract

This systematic review investigates the application of federated learning in mental health and human activity recognition. A comprehensive search was conducted to identify studies utilizing federated learning for these domains. The included studies were evaluated based on publication year, task, dataset characteristics, federated learning algorithms, and personalization methods. The aim is to provide an overview of the current state-of-the-art, identify research gaps, and inform future research directions in this emerging field.

## 1 Introduction

According to the World Health Report 2022 (1), 13% of the global population is living with mental disorders. Furthermore, a significant portion of the population (41% in 2023) experiences elevated levels of stress.^1^ These statistics highlight the urgent need for improved mental healthcare and tools that can enhance both mental well-being and overall quality of life. To address this need, new digital tools are being developed, particularly those leveraging machine learning (2), which show promise as personal diagnostic aids. However, machine learning algorithms, especially modern deep neural networks, are highly data-intensive, requiring personal data from numerous participants. This raises significant concerns about the privacy and security of individuals’ data.^2^

While federated learning (FL) offers promising solutions for privacy-preserving machine learning, particularly in sensitive areas like mental health, it also raises critical ethical concerns. Privacy remains one of the most pressing issues, as although FL allows for decentralized data processing, it is still vulnerable to risks such as data leakage or inference attacks. This is especially crucial in mental health contexts, where protecting patient confidentiality is of utmost importance.

To mitigate these privacy concerns, there is a need to move away from traditional centralized learning approaches, where all data is collected in one location. Instead, privacy-preserving collaborative learning methods should be adopted, where data ownership remains with each participant. Federated Learning (FL) is an emerging paradigm in machine learning that enables the training of algorithms across multiple decentralized devices or servers holding local data, without ever exchanging the data itself, thus offering a privacy-preserving collaborative learning framework.

Fairness is another critical issue. Mental health datasets are often biased, either due to under-representation of certain demographics or inherent biases in data collection methods. If unaddressed, these biases may propagate through FL models, leading to inequitable outcomes that disadvantage certain groups. Ensuring that FL frameworks account for fairness throughout the entire process’ from data collection to model deployment’ is essential for ethical applications in mental health.

Moreover, the deployment of FL systems in mental health research raises broader ethical questions about accountability and informed consent. Patients and users must be fully informed about how their data is being used, even in decentralized settings. Researchers and developers must also be responsible for addressing potential biases or inaccuracies in model predictions. As such, ethical frameworks specifically tailored to FL in mental health should be developed, drawing from interdisciplinary research on ethics, technology, and mental health (3–5).

Federated learning (FL) has the potential to significantly impact real-world applications, particularly in mental health care, where privacy and data security are paramount. As digital mental health platforms such as telemedicine and remote monitoring apps become more widespread, FL provides a way to enhance these systems while ensuring patient privacy. For example, in telemedicine, FL can be used to collaboratively train machine learning models on decentralized data from various healthcare providers, improving diagnosis and treatment recommendations without compromising patient confidentiality. Similarly, in remote mental health monitoring apps, FL allows personalized models to be trained directly on a user’s smartphone or wearable device, ensuring that sensitive data never leaves the device. These applications not only safeguard privacy but also enable more accurate and adaptive mental health care solutions tailored to the individual needs of patients.

**Figure 1:**
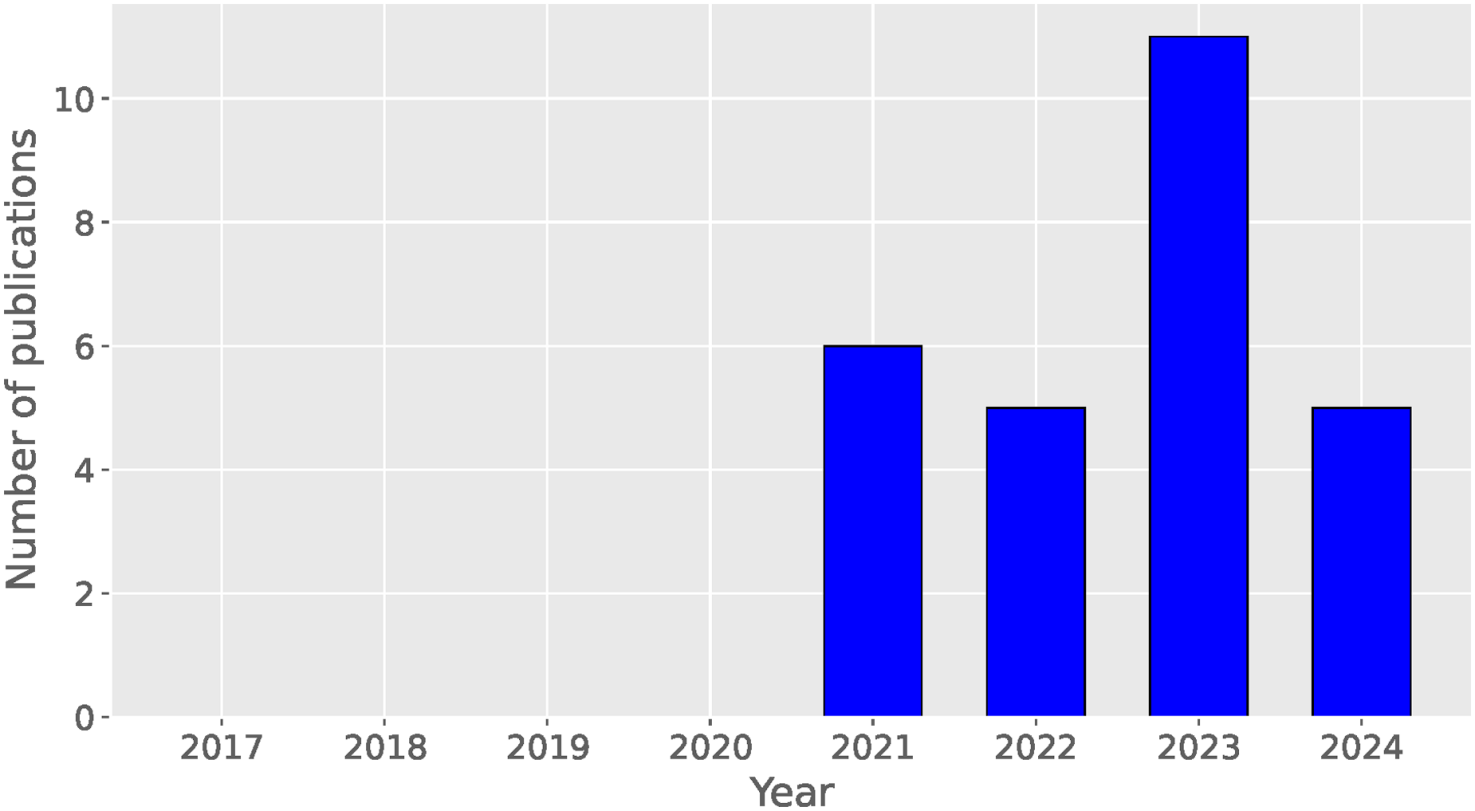
The number of publications for each year since the introduction of federated learning in 2017.

Despite its potential, the application of federated learning in mental health and human activity recognition is still in its early stages, as indicated by the relatively limited number of publications (see Figure 1). A comprehensive understanding of the current state, challenges, and future prospects of federated learning in these domains is therefore essential. This systematic review aims to synthesize the existing literature on the application of federated learning in mental state detection and human activity recognition, critically evaluate the methodological quality of the studies, and identify gaps and potential directions for future research.

Our review is guided by specific research questions, structured around four key perspectives, which form the foundation of our investigation:

1. Demographic and metadata:
  (a) When was the paper published?
2. Input questions: Focus on the topic at hand and input data:
  (a) What is the task at hand?
  (b) What dataset was used?
  (c) Which features were considered?
3. Methodological questions: Model architecture and federated learning:
  (a) Which federated algorithm was considered?
  (b) How are issues of statistical heterogeneity and personalization addressed?
4. Evaluation questions: The results found in the paper:
  (a) How did the model and FL framework perform relative to centralized and local learning?

## 2 Methods

### 2.1 Study design

**Figure 2:**
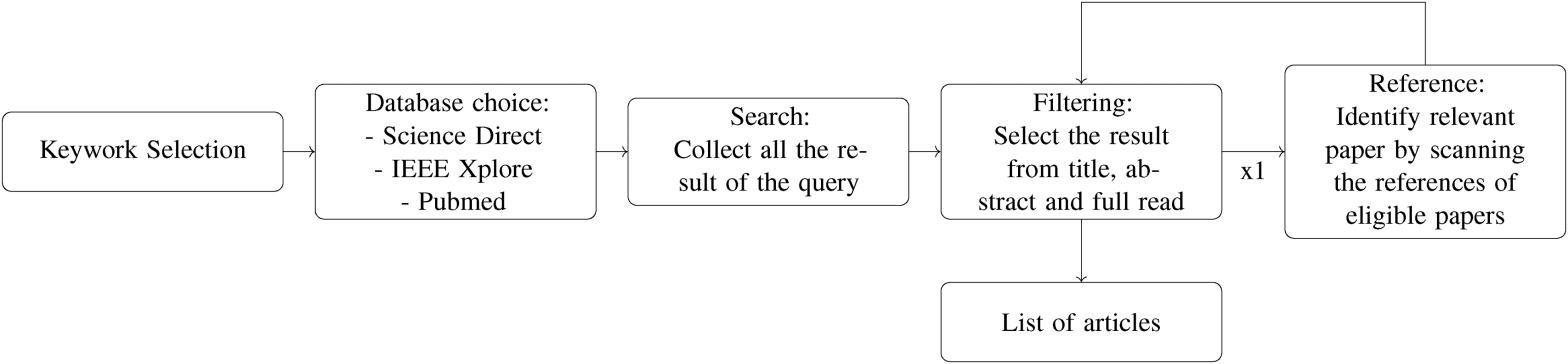
Search method: for each keyword and database we collect the result of the query, filter to select the relevant articles, then search and filter the references of the selected results.

**Figure 3:**
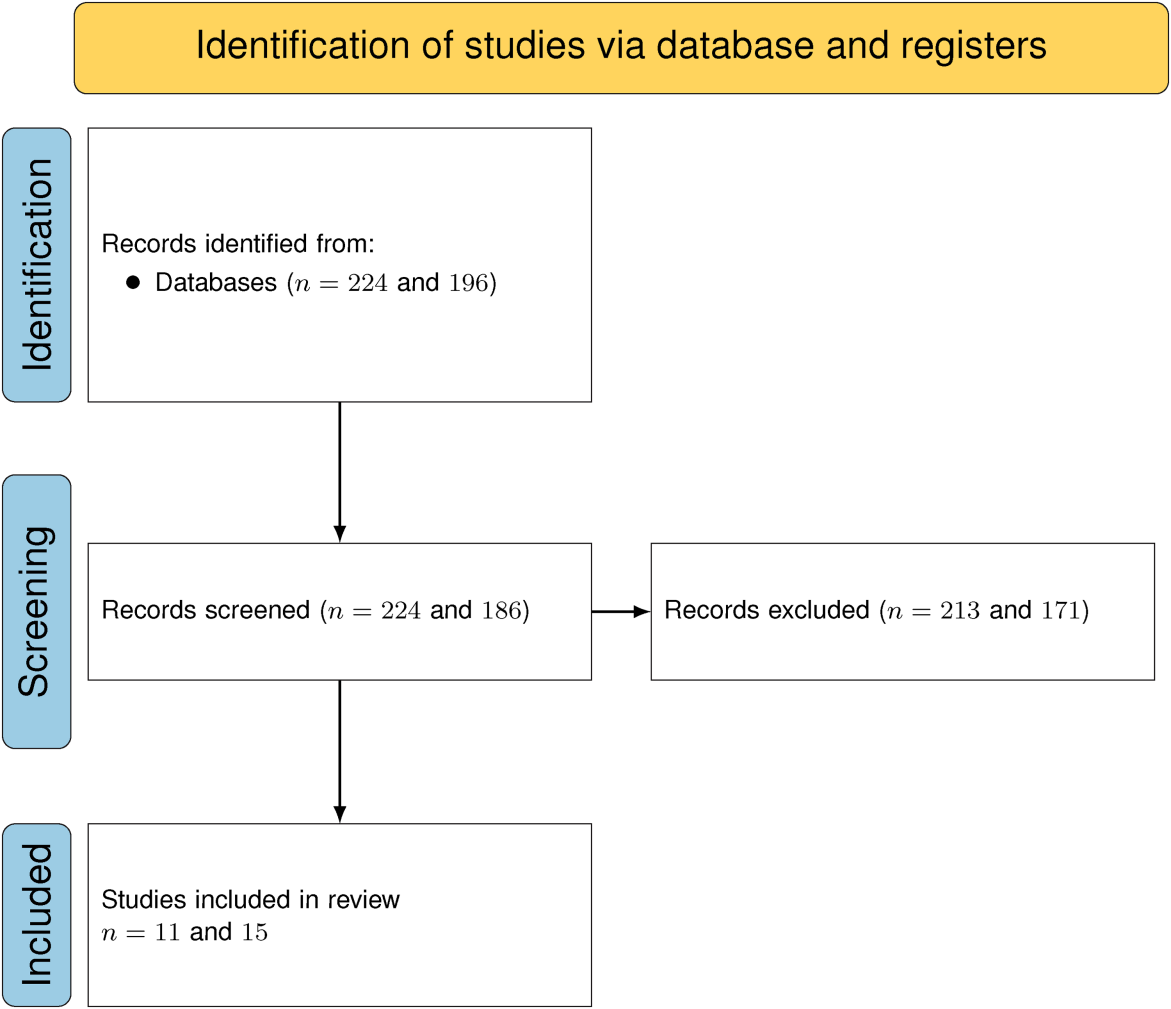
PRISMA flow-chart for both searches.

To answer our research questions, we conducted a structured literature review (SLR) in accordance with the Preferred Reporting Items for Systematic Reviews and Meta-Analyses (PRISMA) guidelines (6) (see Figures 2, 3). This approach involves standardized methods for literature search strategies, as well as clearly defined criteria for the inclusion and exclusion of studies in the final review.

### 2.2 Literature search strategy

We have not set a limited period for our searches since federated learning first appeared in 2017 with the Google research (7). In practice, we will see that the intersection of federated learning and our theme of interest only goes back to 2021 according to Figure 1, making it a rather recent topic. We then conducted a study on privacy-preserving methods for mental health detection and human activity recognition using federated learning. We included the following databases for our searches:

• Science Direct
• IEEE Xplore
• Pubmed

The topic of interest being the application of federated learning for monitoring stress and mental health, our first query was:

“federated learning” AND “stress” AND (“healthcare” OR “health”) AND (“mental” OR “psychology” OR “mind”).

This query gave us 224 results across the three databases we chose, which we filtered using the criteria from Table 1:

**Table 1:** Selection criterion for the first query.

The paper is in English	Inclusion
The paper addresses research on stress, well-being, or mental health	Inclusion
The paper does not use federated learning in the experiment	Exclusion
The paper does not use deep learning	Exclusion
The paper is a duplicate	Exclusion
The paper is a survey article	Exclusion

During the paper selection process, we first excluded papers whose titles were not pertinent to the topic. A further selection was then conducted by reading the abstracts and full texts of the remaining papers. As we reviewed the literature, we observed that the topic of human activity recognition frequently appeared in our results and seemed relevant to the field of mental health monitoring. To ensure comprehensive coverage of this related area, we conducted a second query specifically targeting studies on human activity recognition. The second query chosen was:

“federated learning” AND (“behavior” OR “activity”) AND (“mental” OR “psychology” OR “mind”) AND (“health” OR “healthcare”).

This second query gave 186 results and we filtered the results using the criteria from Table 2:

**Table 2:** Selection criterion for the second query.

The paper is in English	Inclusion
The paper addresses research on human activity recognition	Inclusion
The paper does not use federated learning in the experiment	Exclusion
The paper does not use deep learning	Exclusion
The paper is a duplicate	Exclusion
The paper is a survey article	Exclusion

We repeated the selection process for the second query and then proceeded to scan the references of the articles we had already selected. References were selected using the same criteria as before: first, by identifying titles pertinent to either query and then by matching the criteria outlined in Tables 1 or 2. This process resulted in a total of 27 articles.

## 3 Terminology and pre-requisites

Before discussing the results from the selected papers, we first present the basic concepts and terminology to clarify key aspects of the research field, with a particular focus on federated learning and its specific concepts. For a more in-depth exploration of federated learning, we refer readers to the following sources: (8–12).

In this article, we distinguish three categories of learning approaches: *Centralized learning*, where the data from all participants is collected in a central server to train a model; *Federated learning*, where the data remains local (no sharing of data between participants), but models are shared to enable privacy-preserving collaborative learning; and *Local learning*, where each participant trains their model privately without any collaboration with other participants. These distinctions are essential to understanding the various methodologies applied in the selected studies and how they address the challenges of data privacy and model performance.

### 3.1 Federated learning framework

**Figure 4:**
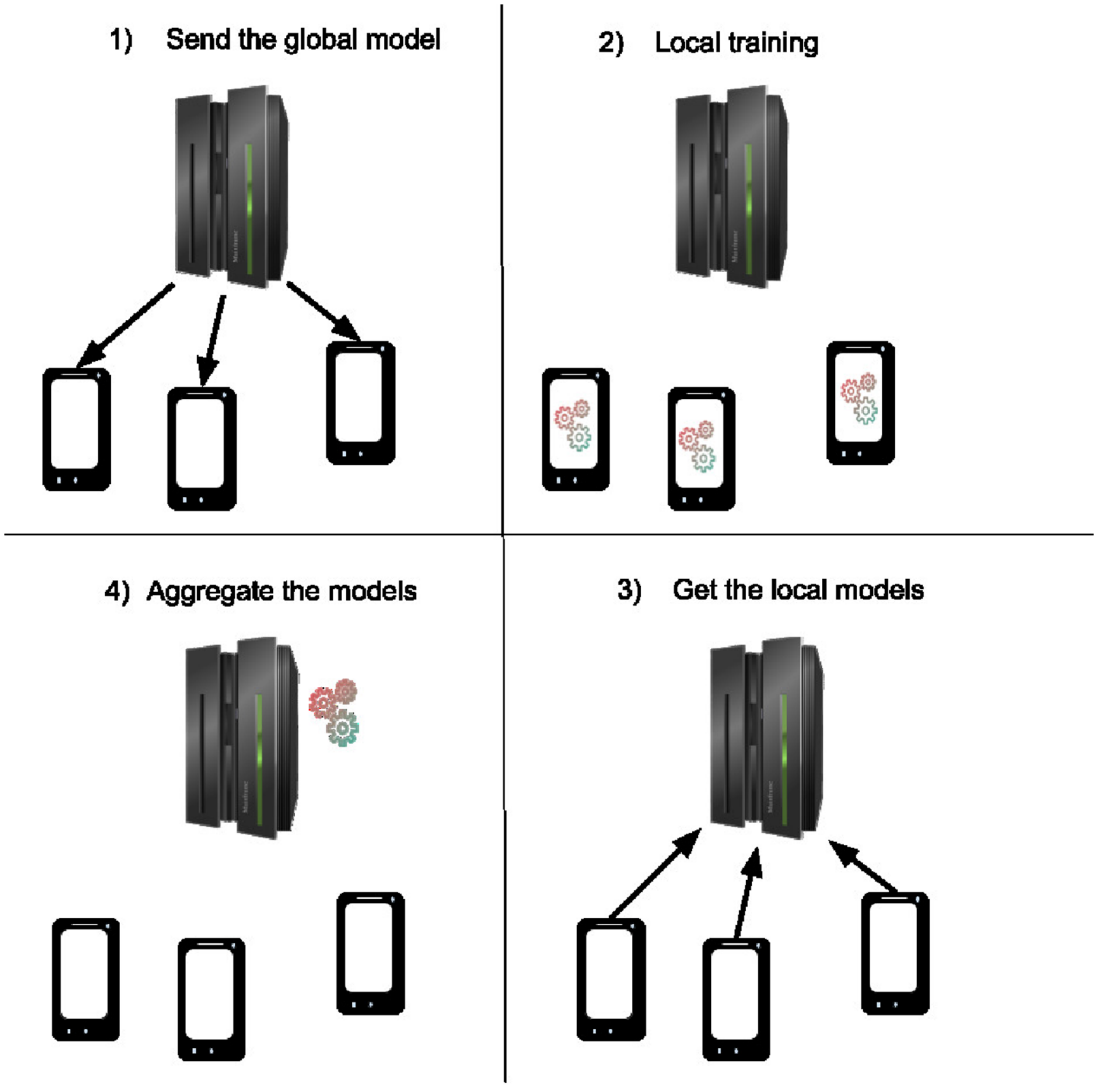
Federated workflow of a training round. The server starts by sending the global model to the clients (1), then the client trains the model with their local data for a few epochs (2) and sends back their new model to the server (3) where these models are aggregated together to obtain a new global model (4).

The general federated learning framework is depicted in Figure 4.

Steps (1) and (3) involve communication rounds, which can be adjusted using mechanisms such as client selection strategies. Step (2) refers to the local training conducted on each client’s device. Step (4) involves the model aggregation mechanism. This aggregation forms the foundation of the federated learning framework known as FedAvg (Federated Averaging).

To illustrate the concept of federated learning, consider a smartphone-based human activity recognition system, where the goal is to develop a model that can predict the smartphone owner’s activity (e.g., walking, running, or sitting) based on sensor data such as GPS, accelerometers, and gyroscopes. In a traditional approach, all data from each user’s smartphone would need to be sent to a central server, where it would be processed to train the model. However, this raises significant privacy concerns, as personal data like location and movement patterns could be sensitive and expose private information.

Federated learning addresses this issue by enabling training to occur directly on each user’s device. Instead of sending raw data to a central server, each smartphone trains its own local model using its sensor data. Then, the smartphone sends only the learned model parameters (i.e., the updates, not the raw data) to a central server. The central server aggregates these updates from all users to improve the overall model without accessing any personal data.

For example, if 10,000 smartphones contribute to the training process, each device sends its updates, and the central server averages them to create a global model that benefits from the collective data. The updated global model is then sent back to the smartphones, and the process continues. In this way, the smartphones collaboratively learn from one another without compromising individual privacy, as the raw data remains on the device.

### 3.2 Data heterogeneity and personalization

One of the main challenges for the generic FedAvg to work well is data heterogeneity. Data heterogeneity refers to the variation or differences in the type, quality, quantity, and distribution of data across different sources or users. In the context of machine learning, it means that data collected from different devices or individuals may reflect individual differences, which can make it harder to train a model that works well for everyone. Data heterogeneity presents two key challenges:

• FedAvg lacks convergence guarantees in the presence of data heterogeneity, which can result in a suboptimal global model or even prevent convergence altogether. To address this, modifications to the framework can be introduced to enhance convergence.
• It is often unrealistic to expect a single global model to perform well across all clients when data heterogeneity exists. Therefore, it is more effective to develop *personalized* models tailored to each client (or group of clients).

To improve convergence, various modifications to the training scheme, as depicted in Figure 4, have been proposed. For personalization, a common approach involves retraining the final global model on local devices, allowing it to benefit from collaborative training while still being adapted to the unique characteristics of each client. For more details on personalization methods in federated learning, see (8, 13). Ultimately, these methods strive to balance collaborative knowledge with personalized insights from each individual’s data by producing a collaboratively informed personalized model.

### 3.3 Privacy mechanisms

Although federated learning ensures that individual client data remains private by not sharing it, certain vulnerabilities still exist, making client information susceptible to various types of attacks, including:

• **Reconstruction attack**: An attacker reconstructs client data using available information, such as communicated model parameters and updates.
• **Inversion attack**: An attacker attempts to reconstruct client data based on the model’s output.
• **Membership-inference attack**: The attacker tries to determine whether a specific sample was part of the training set by analyzing the model’s output.

To mitigate the risks and effectiveness of these attacks, three main approaches are commonly employed:

• **Secure multi-party computation (SMPC)** (14): SMPC enables the secure computation of functions (e.g., sums) on data from different clients without sharing the full data samples. A primary technique is secret sharing, where each client’s data is split into shares that are exchanged among clients. These shares allow computations to be performed without revealing the complete information, as all operations occur on the shares rather than the original data.
• **Homomorphic encryption (HE)** (15): HE is an encryption scheme that allows computations to be carried out directly on encrypted data. For example, operations such as addition or multiplication can be performed within the encrypted space: $HE(w_{1}\cdot w_{2})=HE(w_{1})\cdot HE(w_{2})$, where HE() denotes homomorphic encryption. In the context of federated learning, this technique defends against reconstruction attacks by encrypting all communicated model parameters and/or gradients, enabling aggregation operations in the encrypted space without decryption.
• **Differential privacy (DP)** (16): DP provides a security guarantee by enabling calculations over a dataset while limiting the information that can be inferred about specific samples within that dataset. This is achieved by adding noise to data points, such as to data samples or transmitted model weights in federated learning. The degree of noise can be adjusted, allowing for a trade-off between privacy guarantees and model accuracy. For a detailed overview of differential privacy, see (17).

### 3.4 Ethical concerns

Given the sensitive nature of mental health data, the ethical implications of using federated learning (FL) in this context deserve significant attention. As we have seen, additional measures to protect privacy may be necessary. However, federated learning is also subject to further ethical concerns such as biases and accountability.

Bias in data collection presents a significant ethical challenge. Mental health datasets may underrepresent certain demographics or populations, leading to biased predictions that could disproportionately harm marginalized groups. This imbalance can result in inequitable mental health interventions, further exacerbating disparities in healthcare. Federated learning systems must integrate fairness into their design, ensuring that models perform equitably across diverse populations and addressing biases at both the data collection and model training stages. Methods such as clustering can significantly improve the equity of collaborative training, ensuring that all participants benefit equally.

Another critical ethical issue is the potential impact of inaccurate mental health predictions. Incorrect diagnoses or predictions can have serious consequences for individuals, especially those in vulnerable groups. It is essential to prioritize model validation and include mechanisms for accountability and transparency in FL applications to minimize harm.

To support these efforts, established ethical frameworks, such as the World Health Organization’s (WHO) guidelines on ethics and governance for artificial intelligence in health (18), should be integrated. These guidelines emphasize the importance of transparency, fairness, and accountability in AI systems, all of which are crucial when deploying FL in mental health settings. By adopting these frameworks, FL applications can ensure responsible and ethical use, protecting individuals while promoting innovation in mental health care.

## 4 Results

In this section, we present the results of our systematic review on the application of federated learning in mental state detection and human activity recognition. We begin by summarizing the key findings from the selected studies, highlighting common datasets, methodologies, and outcomes. The results are organized to provide a comprehensive overview of how federated learning techniques have been applied in these domains, including insights into the performances of different methods. Through this analysis, we aim to identify trends, gaps, and potential areas for future research.

### 4.1 Demographical questions

Figure 1 illustrates the distribution of selected research articles by year, beginning in 2017, the year federated learning was first introduced. The figure reveals that research in this area is relatively recent, with no publications on this topic prior to 2021.

The number of articles published each year shows a general upward trend, reflecting growing interest in the field. This trend is particularly notable considering that additional papers are anticipated to be released throughout the remainder of 2024 (the query was done in July 2024).

### 4.2 Input research question

As outlined in Table 3 our literature search strategy, we conducted two distinct searches: one focused on Mental State Detection (MHD) and the other on Human Activity Recognition (HAR). The following table lists the selected papers and the specific topics they address. Notably, one paper (19) deviates slightly from the main focus of our review, as it centers on data preparation and harmonization for federated learning rather than directly applying it to MHD or HAR.

**Table 3:** This is a table of all the article selected with the task at hand classified in three categories, HAR for Human Activity Detection, MSD for Mental State Detection and Others.

	Title	Task
Li et al. (20)	Meta-HAR: federated representation learning for human activity recognition	HAR
Ouyang et al. (21)	ClusterFL: a similarity-aware federated learning system for human activity recognition	HAR
Gao and Konomi (22)	Personalized federated human activity recognition through semi-supervised learning and enhanced representation	HAR
Uprety et al. (23)	Privacy preserving misbehavior detection in IoV using federated machine learning	HAR
Tu et al. (24)	FedDL: federated learning via dynamic layer sharing for human activity recognition	HAR
Shen et al. (25)	Federated meta-learning with attention for diversity-aware human activity recognition	HAR
Novikova et al. (26)	Analysis of privacy-enhancing technologies in open-source federated learning frameworks for driver activity recognition	HAR
Borger et al. (27)	Federated learning for violence incident prediction in a simulated cross-institutional psychiatric setting	HAR
Chhabra et al. (28)	Privacy enabled driver behavior analysis in heterogeneous IoV using federated learning	HAR
Zhao et al. (29)	FedSup: A communication-efficient federated learning fatigue driving behaviors supervision approach	HAR
Vyas et al. (30)	Federated learning based driver recommendation for next generation transportation system	HAR
Suhas and Abdullah (31)	Privacy sensitive speech analysis using federated learning to assess depression	MSD
Nandi and Xhafa (32)	A federated learning method for real-time emotion state classification from multi-modal streaming	MSD
Li et al. (33)	Intelligent depression detection with asynchronous federated optimization	MSD
Khalil et al. (34)	Federated learning for privacy-preserving depression detection with multilingual language models in social media posts	MSD
Gupta and Khullar (35)	Privacy preserving collaboratively training framework for classification of major depressive disorder using non-IID three channel electroencephalogram	MSD
Cui et al. (36)	Privacy-preserving speech-based depression diagnosis via federated learning	MSD
Ahmed et al. (37)	Hyper-graph attention based federated learning methods for use in mental health detection	MSD
Huang et al. (38)	Federated multi-task learning for joint diagnosis of multiple mental disorders on MRI scans	MSD
Kirsten et al. (39)	Sensor-based obsessive-compulsive disorder detection with personalised federated learning	MSD
Chhikara et al. (40)	Federated learning meets human emotions: a decentralized framework for human-computer interaction for IoT applications	MSD
Jiang et al. (41)	Low-overhead clustered federated learning for personalized stress monitoring	MSD
Javed et al. (42)	Cognitive health assessment of decentralized smart home activities using federated learning	MSD
Hu et al. (43)	Source free semi-supervised transfer learning for diagnosis of mental disorders on fMRI scans	MSD
Liu (44)	Depression clinical detection model based on social media: a federated deep learning approach	MSD
Mateus et al. (19)	Data harmonization and federated learning for multi-cohort dementia research using the OMOP common data model: a Netherlands consortium of dementia cohorts case study	Other

We identified 11 papers related to Human Activity Recognition (HAR) and 14 papers focused on Mental State Detection (MSD), each employing federated learning techniques. These papers utilize various datasets, some of which are shared across different studies. A summary of the datasets used is provided in Table 4 together with a list of links where to find them in Table 5:

**Table 4:** Table listing the dataset encountered in the articles, if they are publicly available, the feature, acquisition method, and the number of articles they have been used in.

Dataset	Papers	Public	Feature list	Acquisition method	Number of publications
Custom	Borger et al. (27), Chhabra et al. (28), Ahmed et al. (37), Shen et al. (25), Mateus et al. (19)	✗	Text, time series		5
Social media	Liu (44), Khalil et al. (34), Li et al. (33)	✓	Text	Social media, reddit, twitter, weibo	3
DAIC-WOZ	Suhas and Abdullah (31), Cui et al. (36)	✗	Audio	Audio recording	2
Depth	Ouyang et al. (21), Tu et al. (24)	✓	Depth video	Depth camera	2
IMU	Ouyang et al. (21), Tu et al. (24)	✓	Accelerometer, gyroscope, magnetometer	IMU	2
HARBox	Ouyang et al. (21), Tu et al. (24)	✓	Accelerometer, gyroscope, magnetometer	Smartphone	2
UWB	Ouyang et al. (21), Tu et al. (24)	✓	UWB	UWB nodes	2
ABIDE I	Huang et al. (38), Hu et al. (43)	✓	Image	fMRI	2
ADHD-200	Huang et al. (38), Hu et al. (43)	✓	Image	fMRI	2
PAMAP2	Gao and Konomi (22)	✓	Accelerometer, angular velocity, magnetometer	3 IMU (chest, hand, ankle)	1
RAVDESS	Chhikara et al. (40)	✓	Audio	Audio recording	1
MODMA	Gupta and Khullar (35)	✓	EEG	EEG	1
DEAP	Nandi and Xhafa (32)	✗	EEG, GSR, ECG, video	EEG, GSR, ECG, video	1
OPPORTUNITY	Kirsten et al. (39)	✓	Accelerometer, gyroscope	IMU	1
USC-HAD	Li et al. (20)	✓	Accelerometer, gyroscope	IMU	1
FER2013	Chhikara et al. (40)	✓	Image	Image	1
PhysioNet	Vyas et al. (30)	✓	Skin conductance rate, EMG, ECG, respiratory rate	Physiological data	1
VeReM	Uprety et al. (23)	✓	Unknown	SIMUlated gps data	1
HHAR	Li et al. (20)	✓	Accelerometer, gyroscope	Smartphone	1
UCI-HAR	Gao and Konomi (22)	✓	Accelerometer, angular velocity	Smartphone	1
CASAS	Javed et al. (42)	✓	Unknown	Unknown	1
UAH-DriveSet	Vyas et al. (30)	✓	Gps, accelerometer, speed, pitch, yaw, roll, latitude, longitude, altitude, video	Vehicle records	1
HCI Lab	Vyas et al. (30)	✓	Gps, accelerometer, speed, pitch, yaw, roll, latitude, longitude, altitude, ECG, heart rate, skin conductance rate, body temperature	Vehicle records, physiological data	1
EyeBlink8	Zhao et al. (29)	✓	Video	Video	1
ZJU	Zhao et al. (29)	✓	Video	Video	1
WESAD	Jiang et al. (41)	✓	Blood volume pulse, ECG, eda, EMG, respiratory rate, body temperature, accelerometer	Wearable sensors	1
ABIDE II	Hu et al. (43)	✓	Image	fMRI	1
COBRE	Huang et al. (38)	✓	Image	fMRI	1
No dataset	Novikova et al. (26)	✓	Unknown	Unknowm	1

**Table 5:** Link to the available datasets.

Link	Names
http://www.robesafe.uah.es/personal/eduardo.romera/uah-driveset/	UAH-DriveSet
https://archive.ics.uci.edu/dataset/226/opportunity+activity+recognition	OPPORTUNITY
https://archive.ics.uci.edu/dataset/231/pamap2+physical+activity+monitoring	PAMAP2
https://archive.ics.uci.edu/dataset/344/heterogeneity+activity+recognition	HHAR
https://archive.ics.uci.edu/dataset/465/wesad+wearable+stress+and+affect+detection	WESAD
https://casas.wsu.edu/datasets/	CASAS
https://dcapswoz.ict.usc.edu/	DAIC-WOZ, DAIC-WOZ
https://fcon_1000.projects.nitrc.org/indi/abide/	ABIDE I, ABIDE I, ABIDE II
https://fcon_1000.projects.nitrc.org/indi/adhd200/	ADHD-200, ADHD-200
https://fcon_1000.projects.nitrc.org/indi/retro/cobre.html	COBRE
https://figshare.com/articles/dataset/USC-HAD/22600903	USC-HAD
https://github.com/pg815/Depression_Detection_Using_Machine_Learning https://github.com/Diego-ds/RedditNet	Social Media
https://github.com/xmouyang/FL-Datasets-for-HAR/tree/main	HARBox, UWB, IMU, Depth, UWB, Depth, HARBox, IMU
https://modma.lzu.edu.cn/data/index/	MODMA
https://physionet.org/content/drivedb/1.0.0/	PhysioNet
https://www.eecs.qmul.ac.uk/mmv/datasets/deap/	DEAP
https://www.hcilab.org/research/hcilab-driving-dataset/	HCI Lab
https://www.kaggle.com/competitions/uci-har	UCI-HAR
https://www.kaggle.com/datasets/haider094/veremi-dataset	VeReM
https://www.kaggle.com/datasets/msambare/fer2013	FER2013
https://www.kaggle.com/datasets/uwrfkaggler/ravdess-emotional-speech-audio	RAVDESS

Our analysis reveals that the majority of papers utilize custom-made datasets, reflecting a tailored approach to address specific research needs. In addition, datasets sourced from social media platforms are frequently employed, highlighting their relevance for understanding human behavior in a digital context. Medical datasets also feature prominently, with 9 out of the 32 datasets used across the reviewed articles being related to medical imaging or physiological measurements, including EEG, fMRI, and other physiological data. This emphasis on medical data underscores the importance of physiological signals in mental state detection and human activity recognition.

We also group the datasets in Table 6 by data acquisition method, showcasing which type of data is the most used in the selected papers.

**Table 6:** This table list the different acquisition methods and how often they are used.

Acquisition method	References	Datasets	Feature list	Frequency
fMRI	Huang et al. (38), Hu et al. (43)	ABIDE I (x2), ADHD-200 (x2), COBRE, ABIDE II	Image	6
Smartphone	Gao and Konomi (22), Li et al. (20), Ouyang et al. (21), Chhabra et al. (28), Tu et al. (24), Shen et al. (25)	UCI-HAR, HHAR, HARBox (x2), Custom (x2)	Angular velocity, Accelerometer, Gyroscope, Gravity, Rotation vector, Magnetometer, Orientation, Temperature, Atmospheric pressure, Humidity, Proximity On change, Position every minute, WIFI network connected and other smartphone monitoring	6
IMU	Gao and Konomi (22), Li et al. (20), Ouyang et al. (21), Kirsten et al. (39), Tu et al. (24)	PAMAP2, USC-HAD, IMU (x2), OPPORTUNITY	Accelerometer, Angular velocity, Magnetometer	5
Video	Zhao et al. (29), Ouyang et al. (21), Tu et al. (24), Nandi and Xhafa (32)	ZJU, EyeBlink8, Depth, Depth, DEAP	Video	5
Audio recording	Suhas and Abdullah (31), Chhikara et al. (40), Cui et al. (36)	DAIC-WOZ (x2), RAVDESS	Audio	3
Physiological data	Vyas et al. (30), Jiang et al. (41)	PhysioNet, WESAD, HCI Lab	Skin conductance rate, EMG, ECG, Respiratory rate	3
Social media	Liu (44), Khalil et al. (34), Li et al. (33)	Social media (x3)	Text	3
EEG	Gupta and Khullar (35), Nandi and Xhafa (32)	MODMA, DEAP	EEG	2
Medical facilities	Borger et al. (27), Mateus et al. (19)	Custom (x2)	Text, time series	2
PHQ-9	Ahmed et al. (37), Shen et al. (25)	Custom (x2)	Text	2
UWB nodes	Ouyang et al. (21), Tu et al. (24)	UWB, UWB	UWB	2
Vehicle records	Vyas et al. (30)	UAH-DriveSet, HCI Lab	GPS, Accelerometer, Speed, Pitch, Yaw, Roll, Latitude, Longitude, Altitude, Video	2
Big 5	Shen et al. (25)	Custom	Big 5	1
ECG	Nandi and Xhafa (32)	DEAP	ECG	1
GSR	Nandi and Xhafa (32)	DEAP	GSR	1
Image	Chhikara et al. (40)	FER2013	Image	1
Simulated GPS	Uprety et al. (23)	VeReM	GPS	1
Unknown	Javed et al. (42)	CASAS	No features	1

**Figure 5:**
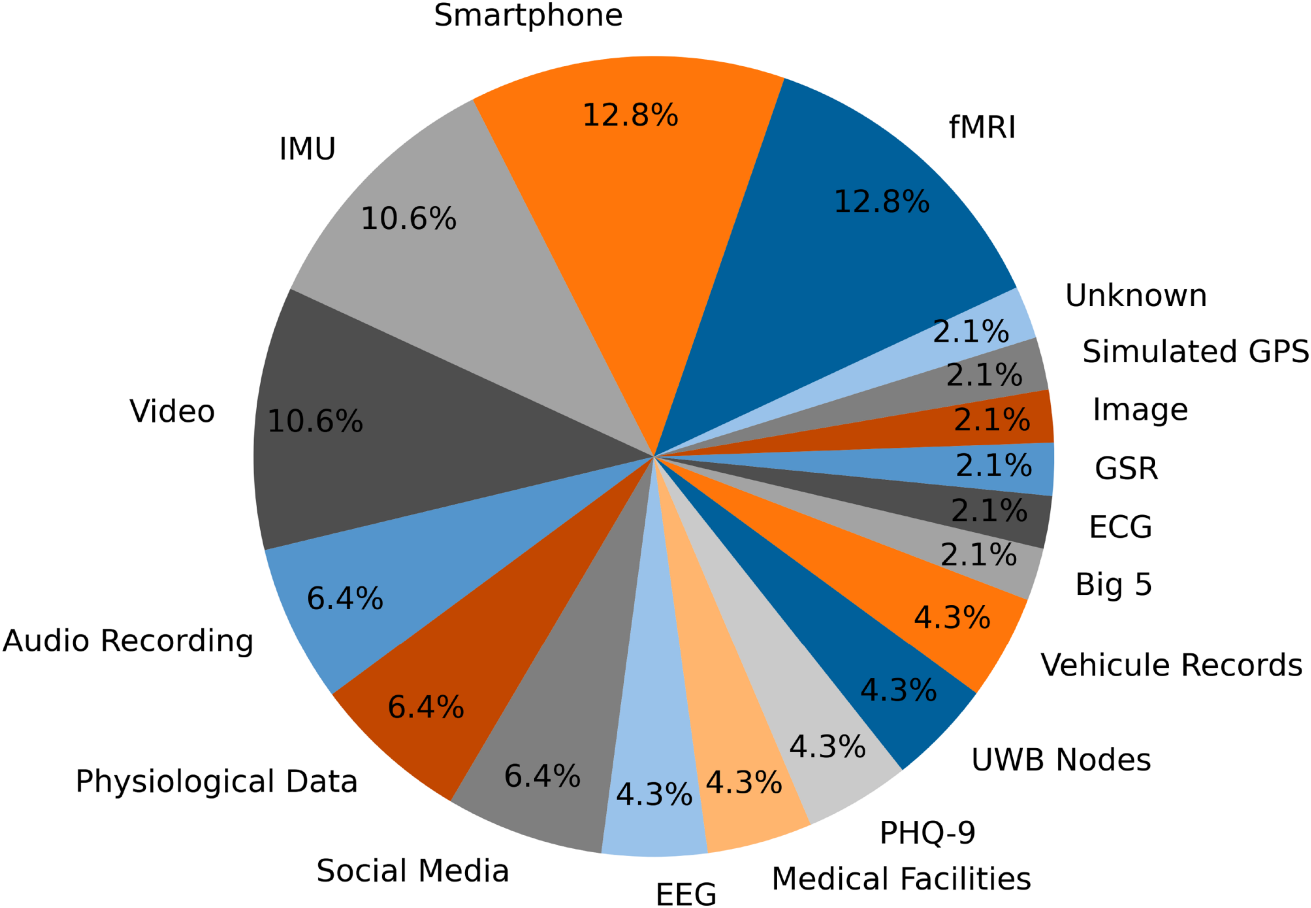
The proportion of the different data acquisition methods used in the selected papers.

Our review indicates (see Figure 5) that the most commonly used data acquisition methods are fMRI and smartphones. Smartphones are particularly popular due to their ability to capture a wide range of features, both from internal sensors (such as accelerometers, gyroscopes, and GPS) and from user behavior patterns. This extensive feature set makes smartphones a versatile tool for gathering diverse data relevant to mental state detection and human activity recognition.

In contrast, while fMRI data is widely used due to its detailed neuroimaging capabilities, it presents challenges in creating personalized datasets. The complexity and cost associated with fMRI scans make it less feasible to develop custom datasets tailored to specific research questions. As a result, most fMRI studies rely on pre-existing datasets rather than generating new, personalized data.

On the other hand, smartphone data is more accessible and adaptable, facilitating the creation of custom datasets that can be specifically designed to address the research objectives. This flexibility allows researchers to gather targeted data that directly supports their study goals.

### 4.3 Methodology

**Figure 6:**
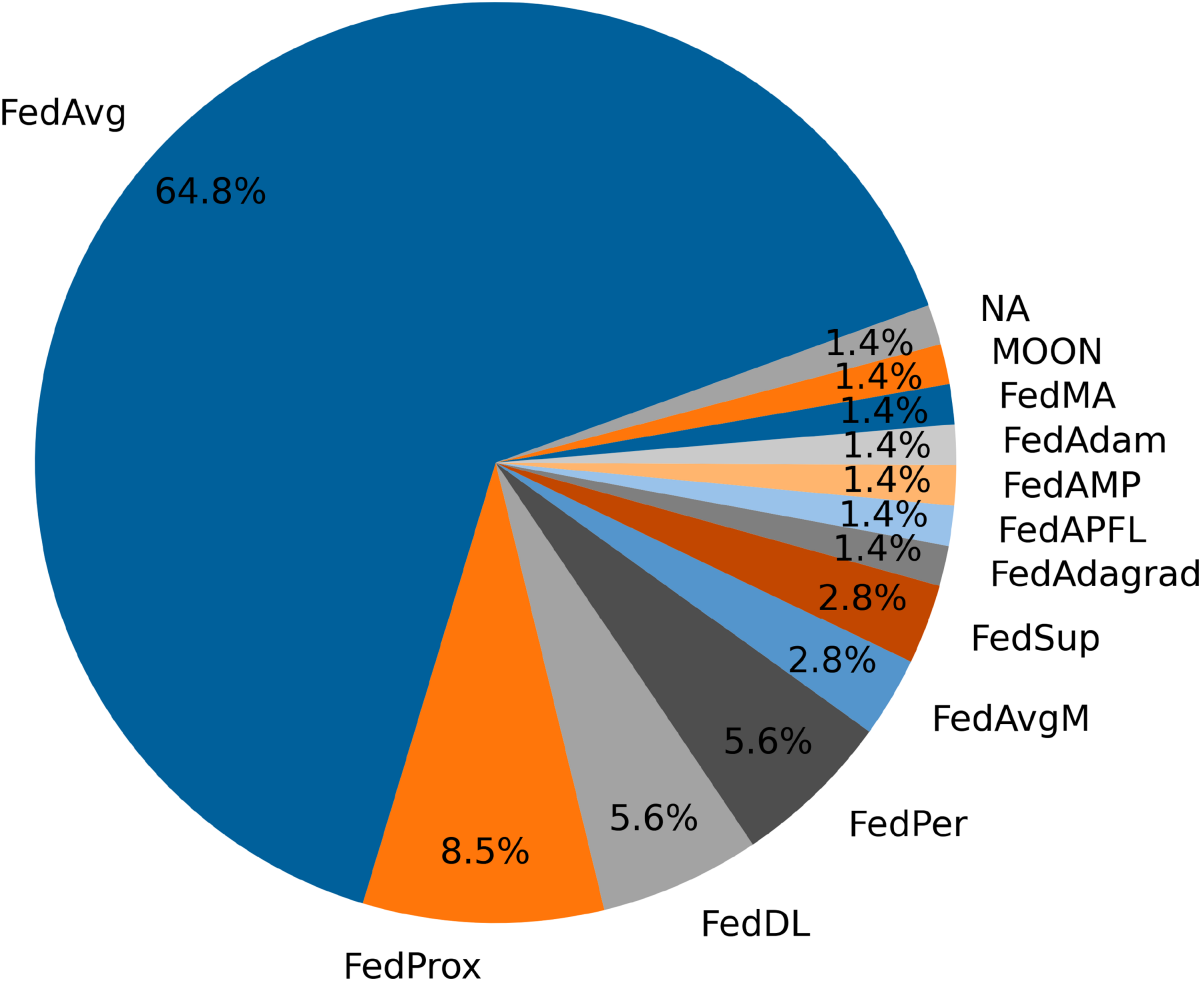
Proportion of the different federated algorithms used in the selected articles.

All of the selected papers employ federated learning methodologies, which encompass a range of algorithms designed to address various challenges in distributed machine learning. Among these algorithms, the basic FedAvg algorithm is by far the most prevalent, representing 64.8% of the federated learning approaches used in the reviewed studies, as illustrated in Figure 6. FedAvg is favored for its simplicity and effectiveness in aggregating model updates from multiple clients, making it a popular choice for a variety of applications within the field.

In this section, we delve into the specific federated learning algorithms and personalization methods employed in the selected studies, providing a comprehensive overview of their applications and performance. We will discuss the relative strengths of different algorithms and explore how various adaptations of federated learning address the challenges within mental state detection and human activity recognition.

In addition to employing federated learning methods, the incorporation of personalization techniques’ discussed in Section 3.2’ is often crucial for achieving effective and accurate learning outcomes. Personalization techniques help tailor the model to individual clients or specific groups, enhancing performance in tasks such as mental state detection and human activity recognition. This importance will be further explored in Section 4.4.

Furthermore, many of the federated algorithms have only been explored by a single paper (44). Specifically, outside of that paper, only one algorithm other than FedAvg has been applied in mental health detection, compared to seven algorithms explored in human activity recognition. This highlights the need for more research on alternative algorithms in mental health detection, especially considering that (44) shows improved performance using these methods.

**Figure 7:**
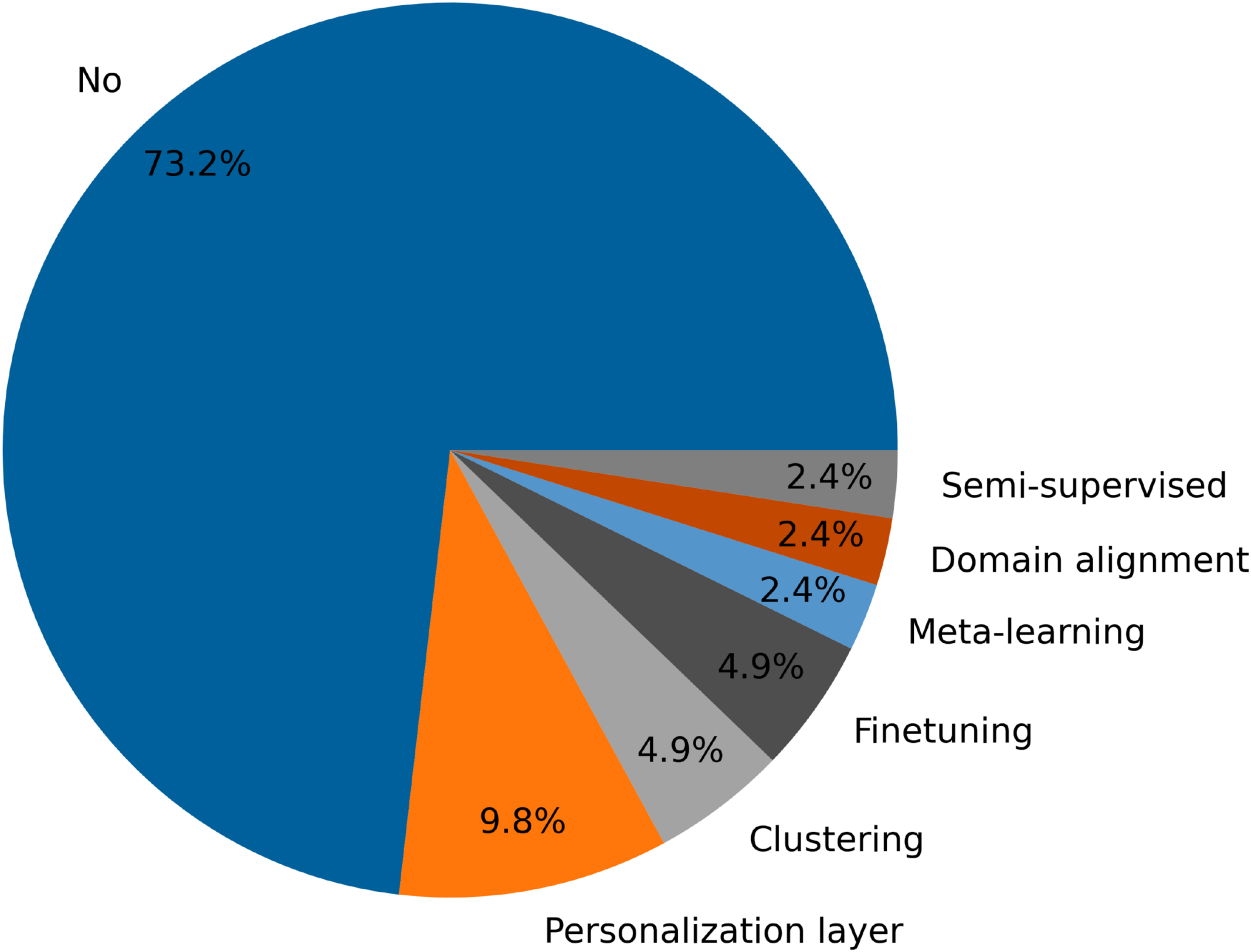
Proportion of selected articles that use personalization methods.

Despite the significance of personalization, Figure 7 reveals that 73.2% of the methods analyzed do not employ any form of personalization. Moreover, a detailed examination of the studies listed in Table 7 shows that 13 out of 24 papers (over half) do not include any personalization techniques. This observation highlights a notable gap in the application of federated learning methods, suggesting that while personalization is recognized as beneficial, it is not yet widely implemented in current research.

**Table 7:** Table describing the federated learning methods used in each selected paper, whether they considered statistical heterogeneity, personalization, and additional security measures.

Paper	Algorithm	Heterogeneity	Personalisation	DP	HE	MPC
Li et al. (20)	FedAvg	✓	Personalization layer and finetuning	✗	✗	✗
Ouyang et al. (21)	FedAvg	✓	Clustering	✗	✗	✗
Gao and Konomi (22)	FedAvg	✓	Semi-supervised and finetuning	✗	✗	✗
Javed et al. (42)	FedAvg	✗	✗	✗	✗	✗
Jiang et al. (41)	FedAvg	✓	Clustering	✗	✗	✗
Chhikara et al. (40)	FedAvg	✗	✗	✗	✗	✗
Kirsten et al. (39)	FedAvg	✓	Personalization layer	✗	✗	✗
Huang et al. (38)	FedAvg	✓	Personalization layer	✓	✗	✗
Ahmed et al. (37)	FedAvg	✗	✗	✗	✗	✗
Borger et al. (27)	FedAvg	✗	✗	✗	✗	✗
Chhabra et al. (28)	FedAvg	✓	✗	✗	✗	✗
Cui et al. (36)	FedAvg	✓	✗	✓	✗	✗
Gupta and Khullar (35)	FedAvg	✗	✗	✗	✗	✗
Hu et al. (43)	FedAvg	✓	Domain alignment	✗	✗	✗
Khalil et al. (34)	FedAvg	✓	✗	✗	✗	✗
	FedAvgM	✓	✗	✗	✗	✗
	FedProx	✓	✗	✗	✗	✗
Li et al. (33)	FedAvg	✗	✗	✓	✗	✗
Nandi and Xhafa (32)	FedAvg	✗	✗	✗	✗	✗
Novikova et al. (26)	FedAvg	✗	✗	✓	✓	✓
Shen et al. (25)	FedAvg	✓	Meta-learning	✗	✗	✗
Suhas and Abdullah (31)	FedAvg	✗	✗	✗	✗	✗
	FedMA	✗	✗	✗	✗	✗
Tu et al. (24)	FedAvg	✓	✗	✗	✗	✗
	FedDL	✓	✗	✗	✗	✗
	FedPer	✓	Personalization layer	✗	✗	✗
	FedProx	✓	✗	✗	✗	✗
Vyas et al. (30)	FedAvg	✗	✗	✗	✗	✗
Zhao et al. (29)	FedAvg	✗	✗	✗	✗	✗
	FedSup	✗	✗	✗	✗	✗
Liu (44)	FedAMP	✓	✗	✗	✗	✗
	FedAPFL	✓	✗	✗	✗	✗
	FedAdagrad	✓	✗	✗	✗	✗
	FedAdam	✓	✗	✗	✗	✗
	FedAvg	✓	✗	✗	✗	✗
	FedAvgM	✓	✗	✗	✗	✗
	FedProx	✓	✗	✗	✗	✗
	MOON	✓	✗	✗	✗	✗

DP, differential privacy, HE, homomorphic encryption, MPC, multi-party secure computation.

**Table 8:** We show the percentage of best and top 2 results for each method characteristic (Centralized, Local, FL, and Personalized). We also include the best percentages when omitting certain methods, such as in the “No Centralized” column, where centralized methods are excluded. Additionally, the table includes the total number of instances of each method across the considered papers.

	Best	Top 2	No centralized	No FL	No personalized	No local	Tot
Centralized	72.7%	81.8%	NA	100.0%	72.7%	72.7%	11
Local	0.0%	0.0%	0.0%	20.0%	0.0%	NA	5
FL	48.7%	94.9%	94.9%	NA	48.7%	51.3%	39
Personalized	80.0%	100.0%	100.0%	80.0%	NA	80.0%	5

Moreover, there is a stark difference in the use of personalization methods between mental health detection and human activity recognition. In mental health detection, most papers do not use personalization methods (with only two algorithms using two different techniques), while human activity recognition employs a broader range of personalization techniques (seven personalization algorithms, including five distinct methods).

Finally, Table 7 provides a comprehensive summary of the federated learning algorithms used in each selected paper. This table details the specific algorithms implemented, assesses whether the studies address the issue of data heterogeneity, and identifies the personalization techniques applied. Additionally, it highlights any additional security measures employed to safeguard data privacy. This summary offers a clear overview of how various aspects of federated learning are handled across the reviewed research, facilitating a deeper understanding of the methodologies used.

Interestingly, some papers fail to mention the issue of data heterogeneity, despite its critical importance in federated learning settings (8, 45, 46). This is notable given the significant impact that data variability can have on model performance and generalization.

Furthermore, the use of additional security measures is relatively rare among the reviewed studies. Differential privacy is the most commonly employed security technique, likely because it is the only security mechanism that can directly affect model performance. The impact of differential privacy on model accuracy is a crucial consideration and explains why it is the most frequently studied privacy mechanism in federated learning. In contrast, other techniques such as homomorphic encryption and secure multi-party computation, while offering strong privacy guarantees, introduce significant computational overhead in a research context and are rarely used.

### 4.4 Performance results

Given the diverse range of topics and datasets covered in the reviewed papers, it is not feasible to make direct comparisons of test evaluation results across different studies. Instead, we adopt a comparative approach within each paper by evaluating the relative performance of different methods. We rank these methods from best to worst based on their performance metrics.

Much of the tasks in the selected papers consist of classification. For example, classifying whether a client is depressed or not, or classifying human activities. Metrics such as accuracy, precision, and AUC are commonly used to evaluate model performance.

For each method analyzed, we classify whether it involves centralized, federated, or local training, and note whether any personalization methods are applied. By focusing on these classifications, we can systematically compare the performance of centralized methods against non-centralized approaches. Specifically, for each type of method (e.g., centralized), we tally the number of times it is outperformed by non-centralized methods. We then compute the percentage of instances where centralized methods are the top-performing learning methods. For example, suppose one article presents the following ranking:

1. Centralized
2. Personalized federated learning
3. Federated learning
4. Local

In this case, centralized learning is the best-performing method. Therefore, we add one to the total number of appearances of the centralized method, and one to the count of times it is the best-performing method. Similarly, for federated learning, it appears twice in the ranking but is outperformed by centralized learning in both cases. Thus, we add two to the count of federated method appearances, but none to the count of times it is the best-performing method. However, we would add two when calculating the ratio of times federated learning is among the top two best methods, instead of just the best. These counts are aggregated for all papers, and the ratios of these totals provide the results we discuss.

Additionally, we calculate the percentage of times each method ranks within the top two methods, meaning they are outperformed by a different method at most once. This approach allows us to gauge the relative effectiveness of centralized methods compared to federated and local methods within the scope of each study.

The results of these comparisons are summarized in the following Table 8, providing a clear overview of how different training approaches perform relative to one another.

In our analysis, we find that centralized learning frequently achieves the best performance, which aligns with expectations. However, in scenarios characterized by high data heterogeneity, personalized methods can sometimes surpass centralized learning. For instance, the study by Ouyang et al. (21) demonstrates that using cluster-based personalization can enhance the performance of centralized learning approaches.

Additionally, when considering the top two results and excluding centralized methods, federated learning (FL) approaches consistently perform as the next best option. This is particularly true when federated methods are combined with personalization techniques, which often elevate their performance to a level comparable to or exceeding that of centralized learning.

In contrast, local training methods consistently rank lower, being at best the third-best option in the performance rankings. This underscores the limitations of local training compared to centralized and federated approaches, particularly in scenarios requiring robust learning from diverse data sources.

Although it is not possible to conduct a systematic performance evaluation due to the variety of tasks and datasets involved, we quantitatively discuss the results of (44), which reports precision, recall, and AUC for local, centralized, and various federated learning methods. Focusing on precision (a similar conclusion can be drawn for other metrics), local training gives 0.788, centralized 0.852, FedAvg 0.818, and their proposed federated algorithm 0.834. This highlights the general trend where federated algorithms perform worse than centralized learning but outperform local training. However, this conclusion is not universal and can be affected by data heterogeneity. For example, in Ouyang et al. (21), they show that using clustering methods (89.06% accuracy) to reduce data heterogeneity produces results comparable to centralized learning (90.83% accuracy), compared to local training (72.19% accuracy) or FedAvg (86.25% accuracy). Nonetheless, even in Ouyang et al. (21), the exact tendencies vary across different datasets, and personalization has varying success compared to local training (though personalized models always outperform FedAvg). This underlines the importance of clustering methods to ensure the benefits of collaborative learning.

## 5 Discussion

In this systematic literature survey, we explored the application of federated learning methods in the domains of mental state detection and human behavior recognition. Utilizing the PRISMA framework (6), we assessed the current state of research and highlighted several key insights.

Our survey reveals that the standard FedAvg algorithm (see Section 3.1) is the most commonly used approach across the studies reviewed. This trend underscores the algorithm’s broad acceptance and its fundamental role in federated learning applications. We have also observed that this is a relatively recent topic that seems to be gaining interest, as shown in Figure 1.

We noted that the majority of datasets employed are public and frequently reused across multiple studies. While public datasets dominate, a few custom datasets have been utilized, reflecting a range of data sources, including fMRI, smartphone data, social media data, and EEG data. This diversity in data acquisition methods highlights the varied nature of the datasets used in the field.

Comparing model performance across studies proved challenging due to the diversity in datasets, tasks, and methodologies. To address this, we ranked methods based on their performance within each paper, revealing that centralized learning generally achieves the best results. This is expected given its ability to leverage comprehensive data. Federated learning methods, particularly when combined with personalization techniques, closely follow in performance. Local training consistently ranks as a less effective method, primarily due to its reliance on limited data from individual clients.

The role of data heterogeneity and personalization in federated learning is significant. Despite their potential to enhance model performance, many studies do not explore the benefits of personalization.

While federated frameworks often prioritize performance, fewer studies incorporate advanced security measures, despite their importance in sensitive domains. Techniques such as homomorphic encryption and secure multi-party computation offer strong privacy guarantees but come with significant computational overhead. These methods can greatly increase processing times and resource consumption, which may not always be justified in terms of performance gains, especially in resource-constrained environments like mobile devices or IoT systems. This trade-off must be carefully considered when deploying FL systems in real-world settings.

As federated learning continues to gain traction in sensitive domains like mental health, addressing ethical concerns is paramount. Fairness and bias remain significant challenges. Mental health datasets often suffer from demographic imbalances, which could lead to biased outcomes if not properly addressed. It is essential that FL models are developed with fairness in mind, ensuring equitable outcomes for all populations. Moreover, informed consent and transparency are crucial when implementing FL in real-world mental health settings. Users should be aware of how their data is being processed, and clear accountability structures must be established to address potential biases or errors in the models.

While FL inherently promotes data privacy by decentralizing data processing, it does not eliminate all risks. Potential vulnerabilities, such as model inversion or reconstruction attacks, must be carefully mitigated to protect individual privacy. Differential privacy, widely adopted for its ability to protect individual data, introduces noise into the training process to obscure personal information. However, this added noise can degrade model accuracy, creating a challenging balance between ensuring privacy and maintaining reliable performance. In sensitive areas such as healthcare, where data confidentiality and model reliability are both critical, this trade-off becomes even more pronounced. Therefore, it is essential to rigorously evaluate the impact of privacy-preserving techniques on model outcomes and explore adaptive approaches that can adjust the level of privacy protection based on the specific requirements of the application. Understanding and addressing these privacy-performance trade-offs is key to achieving practical and secure federated learning systems.

While much research on federated learning has been conducted in controlled settings, real-world deployment, particularly in constrained environments such as mobile devices, wearables, and IoT systems, presents significant challenges that have not yet been fully addressed in the field (47). These devices typically have limited energy resources, meaning that the computational and communication demands of FL could lead to rapid battery depletion. Energy-efficient algorithms, capable of balancing model training and device power consumption, are crucial for ensuring FL’s feasibility in such environments.

Moreover, communication bandwidth is a key limitation. Federated learning relies on frequent exchanges of model updates between devices and a central server, which can lead to substantial network congestion, particularly as the number of clients grows. Techniques that reduce communication overhead, such as compressing model updates or reducing communication frequency, are essential to make FL scalable in real-time scenarios.

Lastly, real-time processing capabilities are constrained by the limited processing power of many IoT and wearable devices. This can impede the ability to quickly adapt models or handle large-scale datasets. Future research should prioritize developing lightweight models and optimizing FL algorithms for resource-constrained devices to support seamless, real-time applications in the real world.

Based on the identified gaps and limitations in the current research, we propose the following areas for future investigation to advance the field of federated learning:

• **Enhanced personalization methods**: Given the inherent heterogeneity among clients, it is crucial to evaluate and apply more consistent personalization methods, enabling models to adapt to individual characteristics. Furthermore, additional questions of fairness, and fair collaborative learning are paramount to ensure that all clients benefit from this framework equitably.
• **Advanced federated learning frameworks**: Although FedAvg remains prevalent, exploring more sophisticated federated learning frameworks could lead to improved collaborative and transfer learning capabilities. Investigating these advanced methods may yield better performance and greater adaptability in various contexts.
• **Research practical implementation solutions**: To enable federated learning in practical situations involving, for example, IoT devices, many additional considerations must be taken into account, including hardware limitations (bandwidth, energy, and computational resources) and privacy assurances in these contexts (accounting for additional computational costs and performance reduction from privacy mechanisms).
• **Dealing with small data samples**: In the field of medical data, clients often have limited personal data, making it important to consider the number of clients and their representativity (fairness). This also makes it challenging to ensure and evaluate the generalizability of the models, as training on small data samples may lead to overfitting, and testing data can be scarce.

These focus areas represent promising directions for advancing federated learning in mental state detection and human behavior recognition, paving the way for more effective and practical applications in these domains.

## Data Availability

The original contributions presented in the study are included in the article/Supplementary Material, further inquiries can be directed to the corresponding author.

## Footnotes

^1^ https://winmr.com/stress-levels-rise-around-the-world/

^2^ https://www.ama-assn.org/system/files/ama-patient-data-privacy-survey-results.pdf
